# Chinese guideline for the diagnosis and treatment of Takayasu’s arteritis (2023)

**DOI:** 10.1515/rir-2024-0002

**Published:** 2024-03-31

**Authors:** Xinping Tian, Xiaofeng Zeng

**Affiliations:** Department of Rheumatology and Clinical Immunology, Peking Union Medical College Hospital, Chinese Academy of Medical Sciences, Peking Union Medical College, National Clinical Research Center for Dermatologic and Immunologic Diseases (NCRC-DID), Ministry of Science& Technology, State Key Laboratory of Complex Severe and Rare Diseases, Peking Union Medical College Hospital, Key Laboratory of Rheumatology and Clinical Immunology, Ministry of Education, Beijing, China

**Keywords:** Takayasu’s arteritis, diagnosis, treatment, guideline

## Abstract

Takayasu’s arteritis (TAK) is a chronic granulomatous inflammatory disease that involves aorta and its primary branches. It is characterized by wall thickening, stenosis/obliteration or aneurysm formation of the involved arteries. In order to standardize the diagnosis and treatment of TAK in China, a clinical practice guideline with an evidence-based approach is developed under the leadership of National Clinical Medical Research Center for Dermatologic and Immunologic Diseases (NCRC-DID). Eleven recommendations for 11 clinical questions that are important to the diagnosis and treatment of TAK are developed based on the latest evidence and expert opinions combined with real clinical practice in China.

## Introduction

Takayasu’s arteritis (TAK) is a chronic granulomatous inflammatory vasculitis that predominantly involves aorta and its major branches. TAK occurs more commonly in young Asian women with the annual incidence approximately 2.6 cases / million people worldwide 90% of patients are younger than 30 at disease onset. The clinical manifestations of TAK includes constitutional symptoms and signs closely related to organ ischemia caused by arterial stenosis or occlusion and aneurysm formation. The current treatment for TAK includes glucocorticoids (GC) combined with traditional synthetic immunosuppressive agents or biological agents. Some patients require vascular interventions or open surgery at some point of their disease. The prognosis is related to the organs involved and the severity of ischemic changes. ^[1]^

The Rheumatology Branch of the Chinese Medical Association published Chinese Guideline for the Diagnosis and Treatment of Takayasu’s arteritis (Draft) in 2004. ^[2]^ In 2008, the European League Against Rheumatism (EULAR) formulated the first recommendations for the management of large vessel vasculitis (LVV),^[3]^ which included TAK and giant cell arteritis (GCA) and was updated in 2018. ^[4]^ The American College of Rheumatology (ACR) also released the guideline for the management of TAK and GCA in 2021.^[5]^ These guidelines has played important roles in guiding the clinical decision-making and standardization of the treatment of LVV. However, the existing guideline have the following problems in guiding the management of TAK in China: (1) Currently, there is no international guideline focusing on the diagnosis and treatment of TAK only. The international guideline for LVV unexceptionally covered both TAK and GCA. The incidence of TAK in China is much higher than that in western countries while GCA is rare in China. In addition, the clinical manifestations and outcomes of Chinese patients with TAK are not exactly the same as those in western countries. (2) The international guideline does not include studies published in Chinese journals in general and lacks evidence from China. Therefore, all recommendations are not fully consistent with the real-world practice in China; (3) The Chinese guideline, Guideline for the Diagnosis and Treatment of Takayasu’s Arteritis (Draft), has not been updated since its release in 2004. During the past two decades, evidences on the diagnosis and treatment of TAK has been published which greatly changed clincial management of TAK. Furthermore, the methodology for guideline formulation has been changed remarkably. So the guideline in 2004 is no longer able to meet the current practical needs for the management of TAK in China. Therefore, the National Clinical Medical Research Center for Dermatologic and Immunologic Diseases (NCRC-DID) (Peking Union Medical College Hospital, PUMCH) collaborated with Vasculitis Study Group of Rheumatologist Association, Cross-Straits Medicine Exchange Association of China, Vasculitis Study Group of Rheumatology and Immunology Physicians Committee, Chinese Medical Doctors Association and Professional Committee of Rheumatology and Immunology of the Chinese Rehabilitation Medical Association, developed the Chinese guideline for the diagnosis and treatment of Takayasu’s arteritis (2023) (hereinafter referred to as this guideline) based on the latest published research work and clinical practice in China. This guideline is also referred to the methods and steps in formulating evidence-based clinical practice guideline.

## Methods

The design and formulation of this guideline strictly follows the WHO handbook for guideline development ^[6]^ and Guiding Principles for Formulating/Revising Clinical Diagnosis and Treatment Guideline in China (2022 Edition),^[7]^ and refers to the items from Reporting Items for Practice Guideline in Healthcare (RIGHT)^[8]^ and Appraisal of Guideline for Research and Evaluation (AGREE II).^[9]^

### 1 Guideline sponsors

This Guideline is endorsed and developed by the NCRC-DID (PUMCH), collaborated with Vasculitis Study Group of Rheumatologist Association, Cross-Straits Medicine Exchange Association of China, Vasculitis Study Group of Rheumatology and Immunology Physicians Committee, Chinese Medical Doctors Association and Professional Committee of Rheumatology and Immunology of the Chinese Rehabilitation Medical Association in the formulation of the guideline. The development of this guideline was launched in June 2022 and finalized in June 2023.

### 2 Guideline working group

As the diagnosis and treatment of TAK involves multiple disciplines, a collaborative and multidisciplinary team was meticulously assembled for the purpose of developing this guideline. Experts in the panel are mainly from the Department of Rheumatology and Immunology, Vascular Surgery, Nephrology, Radiology, Ultrasound, Nuclear Medicine and Evidence-Based Medicine. According to the work they contributed to, they were divided into the guideline steering committee, guideline drafting group, guideline expert panel and evidence evaluation group. Guideline Steering Committee is consisted of 3 academic advisors, 3 expert panel leaders, and 1 chief methodologist. It takes the responsibility of overseeing the entire development process, reviewing the guideline’s full text, and providing expert advice and guidance. Evidence Evaluation Group consists of methodology experts from the Evidence Based Medicine Center of Lanzhou University/Lanzhou University Grading of Recommendations, Assessment, Development, and Evaluations (GRADE) Center. Their crucial role involves conducting comprehensive evidence retrieval, evaluation, and grading. The main responsibility of guideline methodology expert panel is to provide methodological guidance for the recommendation development (including but not limited to evidence retrieval, evaluation, and grading). The Expert Panel is composed of experienced professionals, actively engages in voting on the significance of clinical questions and proposing recommendations. All members of the working group completed a mandatory Conflict of Interest Disclosure form, thereby affirming the absence of conflicts of interest pertaining to this guideline. This transparency ensures their unrestricted involvement throughout the guideline development process.

### 3 Guideline registration

This guideline was registered on the International Practice Guideline Registration Platform (IPGRP-2022CN354), and the corresponding proposal has been uploaded on the Practice guideline REgistration for transPAREncy (PREPARE, http://www.guideline-registry.cn).

### 4 Guideline users and target population

The intended users of this guideline are physicians, integrated traditional Chinese and Western medicine practitioners, nurses, technicians and investigators that may engage in clinic and the scientific research of TAK in hospitals at all levels. The guideline primarily targets patients with TAK.

### 5 Selection and determination of clinical questions

To ensure comprehensive and evidence-based coverage, the writing group solicited valuable inputs from a wide range of experts. with a particular emphasis on evidence generated within China to represent domestic experiences. Additionally, reference was made to guideline and consensuses issued by other countries and international organizations regarding the management of TAK. Following categorization, deduplication, and consolidation, an initial set of 24 clinical questions was formulated based on a comprehensive evaluation of the aforementioned evidence and extensive stakeholder interviews. Subsequently, a Delphi survey was conducted to gauge the perceived importance of each clinical question, utilizing a 7-point Likert scale (ranging from 1 to 7, with higher scores indicating greater importance). New questions were incorporated if they garnered significant attention and recognition by physicians. Finally, a total of 11 key clinical questions were selected for discussion within this guideline, based on a combination of their respective importance rankings and expert opinions.

### 6 Evidence retrieval

The Evidence Evaluation Group meticulously deconstructed the 11 identified clinical questions into their respective Population, Intervention, Comparison, and Outcome (PICO) components before embarking on an extensive search process. Multiple comprehensive databases were searched, including MEDLINE, Cochrane Library, Web of Science, SinoMed, Wanfang database, and China National Knowledge Infrastructure (CNKI). In addition, official websites of reputable organizations such as the National Institute of Health and Clinical Excellence (NICE), the Scottish Intercollegiate Guideline Network (SIGN), the ACR, the EULAR, the Asia-Pacific League of Associations for Rheumatology (APLAR), and Google Scholar were also consulted to augment the search breadth. The references in included literature were manually searched for supplementation. All research was either in Chinese or in English. The research type included systematic review or meta-analysis, randomized controlled trial (RCT), cohort study, case-control study, *etc*. At the same time, manual search was performed on TAK-related guideline and references of included studies. This comprehensive search process encompassed all relevant databases from their inception through March 2023.

### 7 Inclusion and exclusion criteria of evidence

The main inclusion criteria consisted of: (1) Research subjects are patients who met the diagnostic criteria of TAK; (2) No restrictions were placed on the intervention, comparison, or outcome measures; (3) The eligible study designs included systematic reviews, meta-analyses, randomized controlled trials (RCTs), cohort studies, case-control studies, and case series studies. Duplications, conference abstracts, and commentaries were excluded. High-quality systematic reviews were directly included to provide support for the recommendations. In the absence of high-quality systematic reviews, recommendations were based on high-quality RCTs. If both systematic reviews and RCTs were lacking, observational studies would be considered.

### 8 Evidence evaluation and grading of evidence

The evidence evaluation group adopted a measurement tool to assess systematic reviews (AMSTAR),^[9]^ Cochrane tool risk of bias (ROB),^[10]^ quality assessment of diagnostic accuracy studies (QUADAS-2),^[11]^ Newcastle-Ottawa Scale (NOS)^[12]^ for the risk of bias assessment of included systematic reviews and meta-analyses, RCTs, diagnostic accuracy studies, and observational studies, respectively. Two investigators independently performed the assessments, and any discrepancies were resolved through discussion or by consulting a third investigator. The GRADE approach ^[13]^ was utilized to grade the evidence and formulate recommendations (refer to Table 1). For some clinical questions lack of supporting evidence, recommendations were formed based on expert clinical experience, namely Good Practice Statement (GPS).

**Table 1:** Grading of Recommendations Assessment, Development and Evaluation (GRADE)

GRADE rating	Description
Quality of evidence	
High (A)	The authors have a lot of confidence that the true effect is similar to the estimated effect
Moderate (B)	The authors believe that the true effect is probably close to the estimated effect
Low (C)	The true effect might be markedly different from the estimated effect
Very low (D)	The true effect is probably markedly different from the estimated effect
Strength of recommendation	
Strong (1)	The desirable effects of an intervention clearly outweigh the undesirable effects, or clearly do not,
Weak (ss2)	The trade-offs are less certain—either because of low quality evidence or because evidence suggests that desirable and undesirable effects are closely balanced

Note: For some clinical questions lack of evidence, recommendations are formed based on experts’ consensus on clinical experience, which is a good practice statement (GPS).

### 9 Formation of recommendations

The recommendations were formulated by the Expert Panel based on the evidence summarized by the evidence evaluation group. The preferences of Chinese patients, as well as the costs and benefits of the interventions, were taken into consideration. Two rounds of Delphi recommendations surveys were conducted on May 14, 2023 and May 31, 2023. A total of 72 experts were involved. Ninety feedback suggestions were received. From May 2023 online and in person discussions were held in June to get consensus and further modification based on the discussions. All recommendations reached consensus (consensus standard: consensus rate for each recommendation > 85%).

### 10 External review and approval of guideline

The guideline underwent external review by independent reviewers, and revisions were made based on their feedback. Subsequently, the revised guideline were submitted to the steering committee for final approval.

### 11 Dissemination and implementation of the guideline

To ensure that physicians and stakeholders comprehensively understand and appropriately apply the guideline, the guideline working group plans to disseminate and publicize the guideline through various avenues, including (1) introduction in professional journals, websites, and academic conferences, and (2) organizing promotional sessions in some provinces or cities in China to ensure that physicians and other stakeholders fully understand and correctly use this guideline.

### 12 Guideline update

A proactive approach to guideline updates is planned, with a timeframe of 3 to 5 years for revising the guideline. The updates will adhere to international guideline update requirements and guideline.

## Recommendations

### Question 1: How to Diagnose TAK?

**Recommendation 1: For patients with suspected TAK, we recommend adopting the 2022 ACR / EULAR TAK classification criteria for the diagnosis of TAK (1C). Patients with suspected clinical symptoms or signs of TAK should be referred to a team specialized in the diagnosis of TAK led by rheumatologists for diagnostic work-up. Vascular image presentations should take into consideration for the diagnosis and differential diagnosis of TAK (2D). Noninvasive imaging modalities should be considered as the first line choices for the early diagnosis of TAK (1B)**.

The current clinical diagnosis of TAK is mainly based on the TAK classification criteria issued by the ACR in 1990. This criteria set included 6 items: (1) Age at disease onset ≤ 40 years; (2) Claudication of extremities; (3) Decreased brachial artery pulse; (4) Blood pressure (BP) difference > 10 mmHg (1 mmHg = 0.133 kPa); (5) Bruit over subclavian arteries or aorta;(6) Arteriogram abnormality. Arteriographic narrowing or occlusion of the entire aorta, its primary branches, or large arteries in the proximal upper or lower extremities, not due to arteriosclerosis, fibromuscular dysplasia, or similar causes; changes usually focal or segmental. Patients who met three or more of the above six items can be classified as TAK. The sensitivity of this set of criteria was 90.5% and the specificity was 97.8%.^[13]^ However, this classification criteria is not sensitive to the early diagnosis of TAK.

In 2022, ACR/EULAR endorsed a new classification criteria for TAK, which included 2 absolute requirements and 10 additional clinical and image criteria (Table 2). Patients who met the absolute requirements combined with a cumulative score of ≥5 points for additional clinical and image criteria could be classified as TAK. The sensitivity and specificity of this classification criteria were 93.8% and 99.2%, respectively.^[14]^ Chinese investigators also developed a classification criteria for TAK. The sensitivity was 90.63% and the specificity was 96.97%.^[15]^ A study retrospectively analyzed 131 TAK patients and 131 patients with other types of vascular diseases and compared the performance of these two groups of patients between the China criteria, the 1990 ACR classification criteria, and the 2022 ACR/EULAR classification criteria (draft). The sensitivities of these 3 criteria were 85.7%, 47.4%, and 79.1% respectively, while the specificities were 96.2%, 97.7%, and 98.5% respectively. However, the China criteria was not validated in other TAK cohorts.^[16]^ Another retrospective study from China enrolled 97 patients with TAK and 108 patients with large artery stenosis or occlusion caused by atherosclerosis to compare the performance of the 1990 ACR classification criteria and the 2022 ACR/ EULAR classification criteria in the Chinese TAK population. The results showed that the 2022 ACR/ EULAR classification criteria was superior in terms of sensitivity (91.8%), positive predictive value (94.7%), negative predictive value (92.8%), specificity (93.76%) and area under the curve (AUC, 0.979) to the former criteria.^[17]^ Based on the results of above research, we recommend using the 2022 ACR / EULAR criteria to classify TAK patients in China.

**Table 2:** 2022 ACR/EULAR classification criteria for Takayasu’s arteritis

Criterion	Score
Absolute Requirements	
Age ≤ 60 years old at time of diagnosis	
Evidence of vasculitis on imaging	
Classification criteria	
Additional clinical criteria	
Female sex	1
Angina or ischemic cardiac pain	2
Arm or leg claudication	2
Vascular bruit	2
Reduced pulse in upper extremity	2
Carotid artery abnormality	2
Systolic blood pressure difference in arm ≥ 20 mmHg	1
Additional imaging criteria	
One arterial territory	1
Two arterial territories	2
Three or more arterial territories	3
Symmetric involvement of paired arteries	1
Abdominal aorta involvement with renal or mesenteric involvement	3

Note: Patients who must meet the 2 absolute requirements and have a score of ≥ 5 points is needed for the classification of TAK; A is the highest score among the affected arteries in the following nine vascular territories: Nine vascular territories: thoracic aorta, abdominal aorta, bilateral carotid arteries, bilateral subclavian arteries, bilateral renal arteries, mesenteric arteries; 1mmHg = 0.133 kPa.

Most of the clinical symptoms of TAK are non-specific. Once relevant symptoms suggesting TAK occur, clinicians need to carefully differentiate whether the aortic damage is attributed to other causes through imaging examinations. Therefore, the panel recommends that for patients with suspected clinical presentations of TAK, multidisciplinary experts including rheumatologists, radiologists, and vascular physicians should collaborate to improve the accuracy of diagnosis.

With recent development in imaging techniques, conventional catheter-based arteriograms (DSA) have gradually been replaced by non-invasive computed tomography angiography (CTA). In addition, other non-invasive imaging modalities, including color Doppler ultrasound (CDUS), magnetic resonance angiography (MRA), positron emission tomography/computed tomography (PET/CT), can not only reveal the extent and degree of vascular lesions, but also can provide evidence for disease activity. Therefore, these non-invasive imaging modalities have replaced conventional angiography to a certain extent.^[18]^ The diagnostic value of the above-mentioned non-invasive imaging modalities is limited by various factors such as the range and anatomic location of vascular lesions, the operator dependency, accessibility and feasibility. In addition, there is currently no evidence to support the priority of imaging modalities. However, a systematic review in 2018 found that the diagnostic sensitivity of CDUS was 81% (95% CI: 69%–89%) and the specificity was 100% (95% CI not provided). Therefore, noninvasive modalities should be the first line choice for the patients with suspected TAK.

The overall concordance between US and catheter based angiographic findings of vessel stenosis, occlusion or dilation was 86% (95% CI: 75%–92%). The sensitivity of MRA in diagnosing TAK was 92% (95% CI: 88%–95%), the specificity was 92% (95% CI: 85%–96%).^[19]^ A single-center study showed that the sensitivity of CTA in diagnosing TAK was 95% and the specificity was 100%.^[20]^ Another systematic review in 2021, which included 8 cohort and case-control studies, showed that the overall sensitivity and specificity of noninvasive imaging examinations (PET/CT and MRA) for the diagnosis of TAK was 72% and 69% respectively. ^[21]^ Based on these evidence, the expert panel suggested physicians should consider comprehensively about the vascular lesions suspected and the image modalities available in order to select the most appropriate image modalities to facilitate early diagnosis of TAK.

As CTA provides good sensitivity and specificity in identify large artery lesions and is widely available in China, it is the preferred imaging modalities for diagnosing TAK and assessing the extent of vascular lesions and disease severity.

### Question 2: How to Assess Disease Activity and Organ Damage of TAK?

**Recommendation 2: The disease activity of TAK should be comprehensively assessed based on the patient’s clinical symptoms and signs, acute phase reactants and imaging findings. The proposed definition of disease activity by the 2018 EULAR guideline for LVV is preferred for disease activity assessment. NIH/Kerr criteria and ITAS 2010 can also be used to assess disease activity (2D). Takayasu’s arteritis Damage Scores (TADS) is recommended as the instrument to assess organ damage caused by TAK (2C)**.

Disease activity of TAK is defined in both the 2018 EULAR recommendation^[4]^ and the 2021 ACR guideline.^[5]^ The expert panel recommended adopting the definition of the 2018 EULAR recommendation. In 2018 EULAR recommendation for the management of LVV, active disease refers to (1) The presence of typical signs or symptoms of active LVV; (2) At least one of the following: ① Current activity on imaging or biopsy ② Ischemic complications attributed to LVV; ③ Persistently elevated inflammatory markers (after other causes have been excluded). Relapse refers to the recurrence of active disease after remission. Major relapse refers to the recurrence of active disease with either of the following: (1) clinical features of ischemia: stroke, limb claudication; (2) evidence of active aortic inflammation resulting in progressive aortic or large vessel dilatation, stenosis or dissection. Minor relapse refers to the recurrence of active disease, not fulfilling the criteria for a major relapse. Refractory TAK refers to inability to induce remission despite the use of standard care therapy. Remission refers to the absence of all clinical signs and symptoms attributable to active TAK and normalization of erythrocyte sedimentation rate (ESR) and C reactive protein (CRP). In addition, for patients with extracranial disease, there should be no evidence of progressive vessel narrowing or dilatation. Sustained remission refers to remission for at least 6 months with achievement of the lowest GC dose.

In 2018 EULAR recommendation for the management of LVV, key symptoms related to active disease include: (1) New onset or worsening of limb claudication; (2) Constitutional symptoms (*e.g*., weight loss > 2 kg, low-grade fever, fatigue, night sweats); (3) Myalgia, arthralgia, arthritis; (4) Severe abdominal pain; (5) Stroke, seizures (non-hypertensive), syncope, dizziness; (6) Paresis of extremities; (7) Myocardial infarction, angina; (8) Acute visual symptoms such as amaurosis fugax or diplopia. Key findings on clinical examination related to active disease include: (1) Hypertension ( > 140/90 mmHg); (2) New loss of pulses, pulse inequality; (3) Bruits; (4) Carotidynia.

Other assessment instruments for disease activity of TAK includes the National Institutes of Health (NIH) score, the Disease Extent Index in TAK (DEI. TAK) and the ITAS2010/ ITAS. A, and Physician’s Global Assessment (PGA). All these instruments require an comprehensive assessment of the patient’s clinical symptoms and signs, laboratory tests and imaging manifestations, *etc*.^[22,23,24,25]^ According to the Kerr criteria, the presence, recent occurrence or deterioration of at least two of the following four criteria shows active disease: (1) systemic features like fever and arthralgia that cannot be explained by other reasons, (2) elevated ESR, (3) findings of vascular ischemia and inflammation and (4) typical angiographic findings. New onset or worsening of two or more features of TAK are defined as “active disease.” Otherwise, the patients are in remission.^[22]^ The Kerr criteria includes the results of invasive DSA, but physicians often use results of CTA, MRA, or PET/ CT as references when applying Kerr criteria in practice.

DEI. TAK (Disease Extent Index of TAK) derived from Birmingham Vasculitis Activity Score (BVAS), which mainly assesses alterations in clinical symptoms and signs. However, BVAS cannot be well applied to TAK as it was originally developed to evaluate systemic small vessel vasculitis. The total agreement between DEI. Tak and Kerr’s criteria is 94%, while the total agreement between PGA and DEI. Tak is 68%. In addition, the cut-off point of DEI. TAK to discriminate active from inactive disease state has not yet been determined.^[23]^

The India Takayasu Clinical Activity Score (ITAS2010) score was validated in an Indian cohort of 132 TAK patients. It mainly evaluated the symptoms or signs of patients in the past 3 months and the presentations of 6 systems of the body involved. A total score of ≥2 was regarded as active disease (see Table 3 for details). The ITAS. A score combines the ITAS2010 score with acute phase reactants (1 for ESR 21–39 mm/h, 2 for 40–59 mm/h, 3 for ESR > 60 mm/h; 1 for CRP 6–10 mg/dL, 2 for 11–20 mg/dL, and 3 for > 20 mg/dL). ITAS. A score ≥5 is defined as active disease.^[24]^ A Turkish cohort study enrolling 144 TAK patients showed that the total agreement between ITAS2010/ITAS. A and PGA was 66.4% and 67% respectively, and the total agreement between ITAS2010/ ITAS. A and Kerr criteria was 82.8% and 86.3% respectively. Its main limitation is the lack of imaging assessment.^[26]^ In summary, the panel recommended to evaluate disease activity of TAK with the definition proposed by the 2018 EULAR recommendation, while Kerr score or ITAS 2010 score can also be alternative choices.

**Table 3:** Indian Takayasu’s Arteritis Activity Score 2010 (ITAS 2010)

Criterion	Score
1. Systemic symptoms	
1) Malaise/ weight loss >2 kg	1
2) Myalgia/arthralgia/arthritis	1
3) Headache	1
2.Abdomen	
Severe Abdominal Pain	1
3. Genitourinary System	
Abortions	1
4.Kidney	
Hypertension: Diastolic blood pressure >90mmHg	2
Hypertension: Systolic blood pressure >140mmHg	1
5. Nervous system	
1) Stroke	2
2) Seizures (not hypertensive)	1
3) Syncope	1
4) Vertigo/dizzyness	1
6. Cardiovascular system	1
6.1 Bruits	2
1) right carotid artery	1
2) left carotid artery	1
3) right subclavian artery	1
4) left subclavian artery	1
5) right renal artery	1
6) left renal artery	1
6.2 Pulse Inequality	2
BP Inequality	1
6.3 New Loss of Pulses	2
1) right carotid artery	1
2) left carotid artery	1
3) right subclavian artery	1
4) left subclavian artery	1
5) right brachial artery	1
6) left brachial artery	1
7) right radial artery	1
8) left radial artery	1
9) right femoral artery	1
10) left femoral artery	1
11) right popliteal artery	1
12) left popliteal artery	1
13) Right posterior tibial artery	1
14) left posterior tibial artery	1
15) right dorsalis pedis artery	1
16) left dorsalis pedis artery	1
6.4 Claudication	2
1) Arm	1
2) Leg	1
6.5 Carotidodynia	2
6.6 Aortic Incompetence	1
6.7 Myocardial infarction/Angina	1
6.8 Cardiomyopathy/cardiac failure	1

Note: ITAS2010 ≥ 2 points or ITAS.A ≥ 5 points indicates disease activity; Scoring ITAS.A including acute phase response - for ESR, score ITAS plus: 0 for < 20; 1 for ESR 21-39; 2 for ESR 40- 59; and 3 for >60 mm ESR /hr for CRP score ITAS plus: 0 for CRP < 5; 1 for CRP 6-10; 2 for CRP 11-20; and 3 for >20 mg/dl.

Takayasu’s arteritis damage score (TADS) and the Vasculitis Damage Index (VDI) were developed to evaluate disease-related damage in patients with TAK.^[27,28]^ TADS is derived from DEI. TAK and includes 42 items in 7 categories, which evaluate symptoms that last for more than 6 months and do not improve after treatment (Table 4). TADS is correlated with clinical outcomes, such as pulseless, stent patency and mortality. It has been validated in several cohorts at different periods in India. The results of the validation showed that the TADS had a positive correlation with the angiographic progression of TAK and DEI. TAK.^[29,30,31]^ The VDI which contains 64 items, 17 of which are related to TAK, is originally designed to assess organ damage caused by systemic small vessel vasculitis. Few studies using VDI to evaluate organ damage related to TAK and all of these studies focused on pediatric TAK patients.^[32,33]^ There is currently a lack of internationally accepted criteria for evaluating the disease-related damage for TAK patients. Therefore, the experts panel recommends using the TADS to evaluate organ damage of TAK. Furthermore, the expert panel recommends assessing the TAK-related organ damage based on the extent and severityof vascular stenosis, the ischemic manifestations and organ dysfunction. The assessment provides the basis for treatment selection.

**Table 4:** TADS Criteria (Each item below is scored only if it lasts for more than 6 months)

Present	Definition	Score
1. Eyes		
Visual loss in one eye	Visual loss in one eye	1
Visual loss in second eye	Visual loss in second eye	1
2. Chest		
Persistent cough/Dyspnea/Wheeze	Persistent cough, difficulty breathing, shortness of breath	1
Respiratory failure	Incapacitating persistent dyspnoea, which may require oxygen.	1
3. Renal		
DBP > 95 mmHg, SBP > 145 mmHg or requiring antihypertensive medication	Increased blood pressure, such as diastolic blood pressure (>95 mmHg) or systolic blood pressure (>145 mm/Hg)	1
Proteinuria	Albuminuria of more than 1+ on dipstick or > 0.2g in a 24 hour collection	1
Creatinine (>150 pmol/L)	Serum levels by standard lab analysis	1
End stage renal failure	Requires chronic dialysis	1
4. Nervous system		
Organic Confusion/ Dementia	Overt disorientation, loss of memory or prolonged mental reaction time	1
Seizures (non-hypertensive)	Paroxysmal electrical discharges in the brain and producing characteristic physical changes including tonic and clonic movements and certain behavioural changes.	1
Stroke	Cerebrovascular accident resulting in focal neurological signs such as paresis, weakness, etc.	1
2nd stroke	The second cerebrovascular accident resulting in focal neurological signs such as paresis, weakness, etc.	1
Cord Lesion	Transverse myelitis with lower extremity weakness or sensory loss with loss of sphincter control (rectal and urinary bladder).	1
5. Drug-related damages and other damage		
Malignancy	cancer of any organ	1
Infertility	Inability to conceive or deliver a viable fetus	1
Other		1
6. Vascular interventions		
First dilation, stent or surgery	First balloon dilatation, stent implantation or angioplasty	1
Obstruction/restenosis in the above conditions	Obstruction or restenosis of the stent or artery	1
2nd procedure	A second obstruction or restenosis of the stent or artery	1
7. Cardiovascular system		
Bruits	Audible to and fro sound over arteries by auscultation with a stethoscope	1
Pulse and BP inequality	Feeble pulse on one side when compared to a similar pulse on the opposite side. Check for difference in systolic pressure > 10 mmHg between the two limbs	1
Pulse Loss	Loss of previously felt pulse under observation for at least 6 months. Tick box and then move to 7a to record anatomic site(s) involved. Check all of Carotid; Subclavian; Brachial; Radial; Femoral; Popliteal; Posterior tibial; Dorsalis pedis.	1
	7a:	
	left carotid artery	1
	right carotid artery	1
	left subclavian artery	1
	right subclavian artery	1
	left brachial artery	1
	right brachial artery	1
	left radial artery	1
	right radial artery	1
	left femoral artery	1
	right femoral artery	1
	left popliteal artery	1
	right popliteal artery	1
	left posterior tibial artery	1
	right posterior tibial artery	1
	left dorsalis pedis artery	1
	right dorsalis pedis artery	1
Claudication	Pain during movements or activity. Tick box and move to 7b to record site in arm or leg. Exercise-related neck pain or subclavian steel may also be recorded here as claudication	1
	7b: Pain during movement or activity in the arms or legs	1
Aortic Incompetence	Leakage of the aortic valve detected clinically or by ECHO cardiography	1
Ischemic cardiac pain	Chest pain during exertion, relieved by rest or trinitrin	1
Congestive heart failure	Fluid retention with swelling in the feet/body, associated with basal lung crepitations and elevated JVP due to pump failure	1
Cardiomyopathy	Dilated cardiomyopathy	1
8. Other damage items	Any items related to Takayasu’s arteritis, or to treatment lasting more than 6 months, can be recorded here	1

### Question 3: How to Choose Imaging Modalities to Assess Disease Activity in TAK?

**Recommendation 3: Ultrasound/contrast-enhanced ultrasound, CTA, MRA and 18-flurodeoxyglucose (^18^F-FDG) PET/CT can be used to evaluate the disease activity of TAK. The selection of imaging modalities should be based on comprehensive consideration of the anatomic locations and extent of vascular lesions and the accessibility (2C). CTA or MRA is recommended to evaluate the severity and extent of involved arteries (2C)**.

Choosing the right imaging modalities are crucial to the evaluation of disease activity in patients with TAK. Each imaging modalities has its own strengths and weakness and cannot be replaced each other in some extent.

CDUS has the advantages of being non-invasive, non-radiative, widely accessible and repeatable. Thus, it can be used for the assessment of changes of arterial lesions and treatment efficacy.^[18]^ CDUS cannot only be used to measure the mural thickness but also semi-quantitatively measure the disease activity of the of the affected arteries.^[34]^ Indian investigators developed the CDUS-K scoring system (The CDUS TAK score from Kolkata) to score the lesions of 19 arteries in patients with TAK. Even though this scoring system has been demonstrated a high degree of correlation with conventional angiography (0.725, 95% CI: 0.488–0.961) and a moderate correlation with the ITAS 2010 score (*r* = 0.714), follow-up data are lacking.^[35]^ As the neovascularization of vasa vasorum in the aorta is the basis of pathology of TAK, the newly formed micro-vessels in the walls of the aorta and its branches are related to disease activity of TAK. The increase in the number of newly formed micro-vessels can be observed in patients with the active disease compared with that in patients with inactive disease.^[36]^ Contrast-enhanced ultrasound (CEUS) can predict the formation of new vasa vasorum through the contrast agent enhancement in the arterial wall, and can be semi-quantitatively graded according to the degree of contrast agent enhancement. Most investigators graded the intensity of the arterial wall enhancement into three grades. In Grade 1, there is no microbubble contrast in the common carotid arteries, indicating inactive disease. In Grade 2, there is limited or moderate microbubble contrast in the common carotid arteries. The disease activity of patients with Grade 2 enhancement should be assessed combined with clinical presentations. In Grade 3 enhancement, there is extensive wall vascularization. Grade 3 indicates active disease. Studies have also demonstrated the improvement in the CEUS grade after effective treatment, so it can be used for dynamic assessment of treatment efficacy.^[37]^

Several studies explored the diagnostic performance of CEUS in assessing TAK disease activity. The results found that CEUS grade was positively correlated with ITAS 2010 score, NIH score, ESR or CRP, however, there is discrepancy in the strength of the correlation in different studies.^[38,39,40,41]^ A Chinese cohort study used CEUS combined with ESR to evaluate disease activity of TAK, the results showed that patients presented with CEUS Grade 2 combined with ESR > 20 mm/h can be used to define active TAK. The sensitivity of this cut-off has the sensitivity and specificity of 81.1% and 81.5%, respectively. Nevertheless, patients with decreased ESR and CRP might have progression of vascular wall inflammation. So CEUS is more sensitive to vascular inflammation than acute phase reactants.^[42]^ However, due to gas interference and the influence of subcutaneous fat, CDUS has limitations in detecting the descending thoracic aorta and distal aortic branches. In addition, the value of CEUS on assessing disease activity in patients without carotid artery involvement remains unknown. Furthermore, operator dependent of CEUS limits its value in assessing disease activity.

CTA can clearly display mural thickness and luminal changes of the aorta and its main branches. Mural enhancement and ring-like low-attenuation indicate active disease.^[43]^ CTA enables accurate measurement of lumen diameters, and coronary CTA can be used to evaluate the coronary artery involvement in patients with TAK.^[44]^ CTA can clearly detect the changes of coronary arteries if coronary artery involvement is suspected.^[20,45]^ A retrospective case-control study found that median CT density values of pericoronary adipose tissue (PCAT) can be used to assess coronary artery disease activity in patients with TAK (AUC 0.82, 95% CI: 0.70–0.92), although this has not been validated in other studies.^[46]^ The major limitations of CTA are radiation exposure and iodine contrast agent is required. So it is not suitable for routine follow-up of the disease. MRA has a higher resolution of vascular tissue and can reveal the degree of arterial mural thickness, wall edema and lumen changes than CTA. Combined with delayed wall enhancement, it can semiquantitatively evaluate mural inflammation, which is helpful for the comprehensive evaluation of TAK disease activity.^[47]^ The performance of CTA in assessing disease activity of TAK is similar to MRA. The scanning time of CTA is shorter and it allows direct visualization of subtle anatomical structure of arteries when compared to MRA. As shown by multiple studies, CEMRA (contrast-enhanced MRA) scoring system which grades arterial stenosis, wall thickness, and enhancement intensity (levels 1–3) could reflect disease activity of TAK, and are positively correlated with NIH score, ITAS 2010 score, CRP, ESR and other disease activity indicators.^[48,49,50,51]^ MRA is neither invasive nor radiative. Therefore, MRA is recommended by EULAR guideline as the preferred imaging examination and can be used for long-term follow-up. Its limitations mainly include long acquisition time and high cost. It cannot be used for patients with metals embed in body. MRA is also operator-dependent. As inflammation of arterial wall stimulates consequent pathological process such as fibrosis and calcification, which in turn lead to stiffness and stenosis of the arteries involved, so CTA or MRA may be biased when assessing of the locations of stenotic lesions and the efficacy of treatment. In summary, the expert panel recommend that CTA or MRA should be used to assess the extent and severity of lesions of the involved arteries. For patients with contraindications to ionizing radiation and iodinated contrast agents, MRA is preferred.

^18^F-FDG pistron emission tomography/computed tomography (PET/CT) has been used for the assessment of TAK in recent years. It can not only detect the early inflammation of the vessel wall and assess the extent of vessel involvement, but also can reflect the severity of vascular inflammation.^[52]^ Maximum standardized uptake value (SUV_max_) of the lesions on the PET image reflects the intensity of the infiltration of inflammatory cells. The SUV_max_ of the lesions in the vessel wall increases significantly in patients with active TAK, while the SUV_max_ remains stable in patients with inactive TAK.^[53]^ The PET Vasculitis Activity Score (PETVAS) assesses 9 specific vascular segments (ascending aorta, aortic arch, descending thoracic aorta, abdominal aorta, innominate artery, right/Left carotid artery, right/Left subclavian artery) to score the ^18^F-FDG uptake, which has good performance on distinguishing active lesions from inactive lesions.^[54]^ Two cohort studies in China conducted PETVAS validation on TAK patients and found that PETVAS has potential advantages in qualitatively and quantitatively assessing disease activity compared with simple SUV_max_, and PETVAS has a higher correlation with ITAS-2010 scores.^[55,56]^ Although some studies found a correlation between PET/CT and therapeutic efficacy, there is controversy. PET/CT is not satisfactory in displaying microvascular structures due to its low spatial resolution. The application of PET/CT examination is constrained by its high cost and ^18^F-FDG uptake can be affected by some therapeutic drugs, GC in particular. PET/MRI combines PET and MRI, so it has higher tissue resolution than PET/CT but with low radiation dose. However, there are currently few studies on PET/MRI. Large-sample studies are needed to validate to efficacy of PET/MRI in patients with TAK in the future.^[57]^

### Question 4: What Are the Principles and Goals of Treatment of TAK?

**Recommendation 4: The principle of the diagnosis and treatment of TAK is to diagnose and initiate individualized treatment early based on comprehensive assessment of the disease. The short-term treatment goal is to control active disease and achieve clinical remission, while the long-term treatment goal is to prevent and reduce relapses and achieve long-term sustained remission. A multidisciplinary collaborative team led by rheumatologists can prevent and reduce the risk of organ damage and complications, improve prognosis and health-related quality of life**.

High disease activity at early stage increases the risk of organ damage, including complications such as hypertension, arterial stenosis/occlusion, heart failure, cerebral infarction, renal failure *etc*. Therefore, early diagnosis and comprehensive evaluation are beneficial for disease control and improve prognosis.^[58]^ Cohort studies with long-term follow up of patients with TAK demonstrated that hypertension and disease activity are independent risk factors adversely affect the prognosis of TAK.^[59,60]^ Currently, the treatment medications for TAK include GC, immunosuppressive agents, and biologics. These medications have different treatment efficacy and adverse reactions. Therefore, a comprehensive evaluation of the disease should be done based on each patient’s conditions. Individualized treatment plan should be developed based on disease evaluation and taken the efficacy, safety and cost of medications into consideration. Relapses are common in TAK. Studies reported that 50%–96% of patients with TAK have experienced disease relapse within 5 years.^[58,61,62]^ Relapse is a sign of increased disease activity and is the major cause of organ damage and poor prognosis. High-risk factors for relapse include short disease duration, history of TAK relapse, history of cerebrovascular events, renal hypertension, aneurysm, involvement of the ascending aorta and aortic arch, involvement of more than 6 arteries, and elevated acute phase reactants at baseline.^[63]^ Some patients with TAK need to have endovascular therapy or open surgery to improve tissue ischemic changes. Therefore, a multidisciplinary collaborative team work for the long-term management for patients with TAK is recommended. A multidisciplinary team is an effective approach to prevent and control organ damage and complications and to achieve the long-term goal of TAK management.

### Question 5: How to Induce Remission for New Onset Active TAK?

**Recommendation 5: Glucocorticoid is the first line therapy for TAK. For new onset active TAK, prednisone 40–60 mg/day (or equivalent dosage of other GCs) taken orally are recommended (1C). Conventional synthetic disease-modifying antirheumatic drugs (csDMARDs) should be added to GC as remission induction therapy (1A)**.

2018 EULAR and 2021 ACR guidelines recommended GC as the first-line therapy for remission induction for patients with newly diagnosed active TAK. For patients with severe disease, oral prednisone 40–60 mg/d (or equivalent dose of other GCs) should be initially used, with the maximum dose not exceeding 60 mg per day. For patients with only a single localized lesion (such as unilateral common carotid artery, subclavian artery involvement, *etc*.), the initial dose of GC should be prednisone 25–30 mg/day. For patients with mild disease activity (*e.g*., patients with systemic symptoms but no limb ischemia), low-dose GC can be considered as the initial treatment.^[4,5]^ For patients with acute severe organ damage, GC pulse therapy may be considered. A meta-analysis of GC monotherapy for TAK in 2021 demonstrated that most patients in acute phase were sensitive to GC. 60% (95% CI: 45%–74%) of patients achieved clinical symptomatic remission with GC alone. 84% (95% CI: 54%–100%) of patients had normalization of inflammatory markers, while only 28% (95% CI: 6%–57%) of patients had stable disease status on angiography.^[64]^ GC alone usually cannot achieve stable efficacy in the long-term. Up to 50%–80% of patients experienced relapse during GC dose tapering with new vascular involvement. Long term GC treatment would bring many adverse reactions.^[62,65,66,67]^ Another meta-analysis in 2021 reported that 64% (95% CI: 47%–80%) of patients with TAK treated with GC combined with traditional synthetic disease-modifying antirheumatic drugs (csDMARDs) had clinical symptom remission, 81% (95% CI: 59%–97%) of patients had stable disease on angiography, 80% (95% CI: 44%–97%) of patients had normalized Inflammatory biomarkers, but 15% (95% CI: 1%–37%) of patients experienced relapse.^[68]^ Therefore, we recommend adding csDMARDs early to the remission induction therapy for new onset active TAK in order to reduce the dosage of GC, control active disease and prevent relapse.

The csDMARDs used to treat TAK include methotrexate (MTX), mycophenolate mofetil, leflunomide (LEF), azathioprine, cyclophosphamide, cyclosporine and tacrolimus, *etc*. Two prospective cohort studies including 223 patients with TAK aimed to compare the efficacy of cyclophosphamide with LEF for remission induction therapy. The results showed that LEF was superior to cyclophosphamide in efficacy and safety.^[69,70]^ Two retrospective cohort studies including 85 patients with TAK aimed to compare the efficacy of MTX with cyclophosphamide. The results suggested that there was no significant difference between the two groups in clinical remission rate at 6 months. One of the studies reported that there was no significant difference in angiography remission between the two groups.^[71,72]^ A cohort study comparing the efficacy of LEF (*n* = 40) and MTX (*n* = 28) in TAK showed that no statistical significant difference in clinical remission rates at 6, 9, and 12 months, no statistical difference in angiography at 12 months with similar relapse rates between the two groups.^[73]^ Another long-term follow-up cohort study including 12 TAK patients in 2016 reported that patients treated with LEF had a lower mean cumulative GC dose (6.3 g) and shorter duration of GC treatment (20.8 months *vs*. 34.1 months) than those treated with other DMARDs (13.3 g).^[74]^ A meta-analysis included 8 uncontrolled observational studies of LEF in patients with TAK. Leflunomide was superior to MTX and cyclophosphamide in remission induction, relapse prevention, and tolerability. Another study showed that the efficacy of LEF was comparable to tocilizumab.^[75]^ Since the studies on csDMARDs for the treatment of TAK are observational studies and lack of high-quality evidence, the expert panel recommend that csDMARDs should be selected based on comprehensive evaluation of the patient’s disease activity, severity, complications or comorbidities, fertility requirements, costs and safety.

### Question 6: How to Induce Remission for Patients with Relapsed or Refractory Disease?

**Recommendation 6: For patients with minor relapse, increase the dose of GC to at least the previous effective dose and adjust the use of csDMARDs is recommended (1C). For patients with major relapse, high-dose GC, or increasing the GC dose as new onset active disease, and modify csDMARDs is recommended (1C). For patients with relapsed or refractory TAK treated with csDMARDs, biological DMARDs should be considered (2C)**.

Relapse of TAK is very common, especially during GC tapering or in patients with GC monotherapy. Relapse may lead to progression of organ damage. As there are currently no studies on patients with relapsed TAK, EULAR / ACR/ Italian guideline recommended to (*i.e*., no symptoms of major relapse), increase the dose of GC to at least the previous effective dose ^[4,5,76]^ and adjust DMARD for patients with minor relapse. For TAK patients with major relapse (clinical manifestations or signs of ischemia, or progression of vasculitis, *etc*.), restarting GC, or increasing the dose of GC as for new onset disease and modifying DMARDs is recommended.

In recent years, some observational studies indicated that biological DMARDs, such as tumor necrosis factor (TNF) inhibitors (infliximab, adalimumab), interleukin (IL)-6 receptor antagonist tocilizumab, anti-CD20 monoclonal antibody rituximab, *etc*. could improve clinical or imaging manifestation in patients with inadequate response to csDMARDs or relapse, however, other studies did not find this favorable efficacy.^[68,77,78,79,80]^ Overall, these observational studies suggested that approximately 66% of patients treated with GC combined with biologic DMARDs achieved remission, but there was no evidence that biologic DMARDs were superior to csD-MARDs. Therefore, biologic DMARDs are recommended as second-line therapy for refractory or relapse TAK after failure of csDMARDs treatment.

Studies on the treatment of relapsed or refractory TAK are very limited. In a meta-analysis including 19 studies that evaluated TNF inhibitors in relapsed or refractory TAK, 81% of patients achieved at least partial clinical response (95% CI: 72%–89%, 15 studies, 208 patients), and 86% of patients achieved angiographic stabilization (95% CI: 74%–95%, 10 studies, 148 patients), and 91% of patients had a reduction in vascular uptake on positron emission tomography (95% CI: 75%–100%, 2 studies, 26 patients), 80% of patients had a decrease in acute phase reactants (95% CI: 56%–98%, 2 studies, 17 patients), 81% of patients had GC tapered to the median dose (95% CI: 61%–95%) and 32% of patients experienced relapses (95% CI: 14%–53%, 6 studies, 87 patients).^[68]^

A RCT study (TAKT) from Japan investigated the efficacy of tocilizumab (TCZ) in relapsed TAK. The result showed that the time to relapse in the tocilizumab group was longer than the placebo group but did not achieve statistical significance.^[81]^ In an open-label study, 36 patients received subcutaneous injection of TCZ weekly. Most patients had clinical improvement and maintained over 96 weeks. They also achieved GC dose reduction without imaging progression.^[82]^ An observational study on patients with refractory TAK indicated that 10 patients who were treated with GC combined with multiple csDMARDs (median duration 27 months) received monthly TCZ infusions. All patients achieved clinical remission (ITAS score of 0), but all relapsed after TCZ withdrawal.^[83]^ A systematic review of TCZ in patients with TAK reported that 87% of patients had clinical response (95%CI: 77%–94%), and 88% of patients had angiographic stabilization (95% CI: 74%–98%), 62% of patients (95% CI: 23%–95%) had reduction of vascular uptake on positron emission tomography and 94% of patients (95% CI: 83%–100%) had a decrease of acute phase reactants, 83% of patients had GC dosage reduction to the median dosage (95% CI: 71%–92%), and 26% of patients relapsed (95% CI: 11%–43%).^[68]^ The results of 2 observational studies assessed the efficacy of biologics in patients with relapsed/refractory TAK showed that there was no significant difference in clinical response, vascular complications and adverse events between tocilizumab and TNF inhibitors.^[84,85]^ A systematic analysis including 35 studies 1082 patients (most patients with moderate to severe disease activity) who were treated with tocilizumab and TNF inhibitors demonstrated similar efficacy between tocilizumab and TNF inhibitors (TNFi). This is contrary to the ACR 2021 recommendations that suggested that TNFi was superior to tocilizumab.^[86]^

Janus kinase (JAK) inhibitors have been widely used to treat inflammatory rheumatic diseases, however, their use in the treatment of refractory and relapsed TAK is still under exploration. A prospective study that included 53 patients with TAK compared the efficacy and safety of tofacitinib with MTX. After 12 months of follow-up, compared with GC combined with MTX, GC combined with tofacitinib was more effective in complete remission (88.46% *vs*. 56.52%, *P* = 0.02), relapses reduction (11.54% *vs*. 34.78%, *P* = 0.052) , decrease of inflammatory markers and better safety profile.^[87]^

A case series reported 16 patients with refractory TAK who were treated with rituximab. The result showed that 50% of patients achieved complete or partial clinical response. Therefore, rituximab can also be used as a second or third-line biologic for treatment of relapsed or refractory TAK.^[88]^

In summary, biologics such as tocilizumab, TNF inhibitors, *etc*. should be considered for patients with relapsed or refractory TAK who failed to respond to csDMARDs. Tofacitinib or rituximab may be used as the second or third-line therapies after failure of tocilizumab and TNF inhibitors.

### Question 7: How to Maintain Persistent Remission?

**Recommendation 7: When patients achieve remission, tapering GC dose gradually to 15–20 mg/day within 2–3 months and to ≤10 mg/day after 1 year is recommended**.

There are currently no studies comparing different GC reduction regimens for patients with TAK. In the TAKT study, GC tapering 10% per week resulted in a high relapse rate (80% at weeks 8–16).^[81]^ In the RCT study on abatacept in patients with TAK, the GC monotherapy group had a similar relapse rate.^[25]^ Therefore, the expert panel recommend that once the active disease is controlled, GC dosage should be tapered to 15–20 mg /day within 2–3 months combined with the maintenance csDMARDs and then tapered more slowly to ≤10 mg/d after 1 year or the lowest dosage possible.

### Question 8: When to Initiate Antithrombotic Therapy in Patients with TAK?

**Recommendation 8: Routine antiplatelet or anticoagulant therapy is not recommended. For patients with high risk factors for organ ischemia, thrombosis, cardiovascular and cerebrovascular diseases or during peri-operative and post-operative period, antiplatelet therapy is recommended (2D)**.

A retrospective cohort study on 48 patients with TAK reported that 29.2% of patients experienced acute ischemic events and antiplatelet therapy had a protective effect against the occurrence of ischemic events (hazard ratio = 0.055, 95%CI: 0.06–0.514), whereas no significant protective effect was observed with anticoagulation therapy.^[89]^ Considering the protective effect of antiplatelet therapy on ischemic events and the potential risk of bleeding, patients with TAK do not need to receive antiplatelet therapy routinely. If patients have organ ischemic complications or cardiovascular diseases such as acute coronary syndrome and acute myocardial infarction, stroke and other high risk factors,^[90]^ or during peri-operative and post-operative period, antiplatelet therapy should be initiated.^[91]^

### Question 9: When Should Patients with TAK Undergo Surgical Intervention and What Is the Perioperative Medical Therapy?

**Recommendation 9: Emergency or confine surgical intervention should be performed for patients who have life-threatening csonditions or severe organ ischemia or complications (1D). Elective surgery should be performed in stable phases of the disease (2C). The time and surgical intervention approach should be a collaborative decision of the multidisciplinary team (2D). Optimized csDMARDs therapy and antithrombotic therapy during the perioperative period can significantly improve outcome (2C)**.

Surgical intervention is an important part of the management of TAK. The main purposes of surgical treatment for TAK patients include relieving organ ischemia, improving hemodynamic abnormalities, improving organ function, alleviating TAK-related complications, and improving the overall prognosis. Surgical intervention can be divided into emergency interventions, confine interventions or elective interventions^[92,93,94,95,96,97]^: (1) Emergency interventions are indicated for life-threatening situations, including acute aortic dissection, threatened rupture of aortic dissection or threatened rupture of aneurysm, acute myocardial infarction. Patients should be referred to the surgical team immediately for emergency surgery. (2) Confine interventions are indicated in conditions when severe tissue and organ ischemia occurred, such as severe cerebral ischemia, myocardial ischemia, other vital visceral ischemia, limb ischemia, refractory hypertension caused by renal artery stenosis or aortic stenosis, and risk of aneurysms rupture, severe aortic regurgitation and other severe valve lesions leading to cardiac insufficiency. Patients with above-mentioned conditions despite adequate medical therapy, confine intervention should be performed. (3) Elective interventions are indicated for patients without the above-mentioned serious life/organ function-threatening conditions in the short term. Elective surgical interventions should be performed with the support of the multidisciplinary team if the patient is still at potential risk of chronic organ ischemia and abnormal hemodynamics caused by arterial lesions. For instance, even though secondary hypertension is well-controlled under medical therapy, long-term renal artery stenosis/occlusion may finally lead to renal atrophy. These patients should be treated with elective surgical interventions.

Several observational studies have shown that the incidence of postoperative complications is significantly related to the inflammation of the targeted arteries during surgical intervention. The incidence of postoperative complications and reoperation rates in patients with active TAK are significantly higher than those in patients undergoing the procedures in disease remission.^[97,98,99,100]^ Therefore, the expert panel recommend that elective surgical intervention in TAK patients should be performed during disease remission.

Surgical interventions for TAK mainly include the following approaches: (1) Open surgery: such as cardiac valve replacement, vascular reconstruction surgery including patch or bypass graft surgery and endarterectomy, *etc*. Vascular reconstruction surgery is the most widely used type of open surgery to treat vascular stenosis. The bypass graft can be artificial blood vessels or autologous blood vessels. The specific surgical approach is chosen based on the anatomic locations and severity of the lesions. (2) Endovascular therapy: mainly including balloon dilatation and endovascular stenting. Taking renal artery stenosis as an example, balloon dilation is the main endovascular therapy. If arterial dissection or elastic recoil occurs after balloon dilation, a rescue stent implantation is usually necessary. (2) Hybrid surgery: It combines endovascular therapy with open surgery.

Both open surgery and endovascular therapy have advantages and disadvantages. Open surgery, such as artificial blood vessel/autologous blood vessel bypass, has been demonstrated to have a high postoperative patency rate and advantages in managing complicated, long-segment and occlusive lesions. However, it is invasive with high rates of perioperative mortality and complications. Endovascular therapy such as balloon angioplasty and stent implantation are minimal-invasive and repeatable with reliable efficacy on simple and short-segment lesions, but the long-term restenosis rate and re-operation rate remains high.^[94,95,101,102]^ A meta-analysis comparing endovascular therapy to open surgery showed that for TAK patients who underwent endovascular therapy, regardless of disease activity, had a significantly higher incidence of postoperative restenosis rate (OR = 5.18, 95% CI: 2.78–9.62, *P* < 0.001), especially when applied to the coronary arteries, supra-aortic branches and renal arteries.^[103]^ In recent years, the application of new balloon dilatation catheters such as drug-eluting balloons in TAK renal artery stenosis has reduced the risk of postoperative restenosis. Comprehensive assessment of disease activity, optimize peri-operative medical therapy can reduce the restenosis rate and improve the outcome of patients with surgical intervention.^[93,104]^ Therefore, the decision for the choice and the timing of surgical approaches should be made based on careful evaluation of various factors, including disease activity, locations and extent of lesions, potential risks and the patient’s general condition. The final decision on the approach of surgical interventions should be made by a multidisciplinary team composed of rheumatologists, vascular surgeons, interventional radiologists, neurologists and anesthesiologists. The final decision should be made by taking the patient’s willingness into consideration.

Perioperative medical therapy is crucial to TAK patients undergoing surgery. Optimized immunosuppressive therapy can significantly improve patients’ outcome. The study by Perera *et al*. showed that even in TAK patients who underwent elective surgery during disease remission, peri-operative immunosuppressive therapy combined with perioperative intravenous hydrocortisone (100–200 mg/day) may improve the outcome of surgical intervention, especially endovascular therapy.^[93]^ For patients who underwent emergency/confine surgery during active phase due to critical conditions, highdose GC should be considered,^[5]^ or GC combined with csD-MARDs or bDMARDs (such as tocilizumab or TNF inhibitors) in order to achieve remission rapidly.^[105]^ Although perioperative immunosuppressive therapy has adverse reactions, they are generally controllable.

Currently, there is lack of high-quality evidence on perioperative antiplatelet or anticoagulant therapy on patients with TAK. Thus, antithrombotic therapy for TAK patients is generally referred to that for vascular surgery. For instance, Sharma and Gupta et al. suggested that TAK patients who scheduled endovascular therapy for renal artery stenosis should start aspirin plus clopidogrel for at least 3 days before surgery and maintain for at least 3 months after surgery. Aspirin or clopidogrel antiplatelet single therapy can be continued 3 months after the procedure.^[106]^ For patients who cannot tolerate dual antiplatelet therapy, antiplatelet single therapy can be considered. If the patient has extensive arterial lesions or venous thrombosis, anticoagulant therapy may be considered. However, as patients with TAK may take high doses of GC or have refractory hypertension when they are operated, so the risk of gastrointestinal and intracranial bleeding is significantly increased. Therefore, the initiation of antithrombotic therapy should be cautious. Preventive treatments should be considered, such as proton pump inhibitors and blood pressure should be strictly controlled.

In addition, other risk factors for vascular disease in patients during peri-operative period should also be strictly controlled, such as hyperlipidemia, smoking, hyperuricemia, *etc*. These measures help to improve patients’ prognosis.

### Question 10: What to Do for the Long-term Management of Patients with TAK?

**Recommendation 10: Long-term follow-up and monitoring of disease progression are warranted for patients with TAK. Patients with active disease should be followed every 1–3 months, while patients with inactive disease should be followed every 3–6 months. Disease activity assessment, monitoring for complications and drug-related adverse reactions are recommended at each follow-up visit (2D). Patient education, encouraging self-management and maintaining healthy lifestyle are recommended (1B)**.

The prognosis of TAK is generally good. The rich collateral circulation formed during the disease process enables blood supply to vital organs. The long-term survival rate is generally high. The 5-year survival rate is 67% –100%.^[59,92,107^] Two long-term follow-up cohort studies showed that the 10- year survival rate of TAK patients ranged from 85% to 96%.^[108,109]^ The prognosis mainly depends on the severity of hypertension and the preservation of vital organs function such as the brain, heart and kidney. The main causes of death of TAK patients include stroke, heart failure, renal failure, postoperative complications and infection.^[4]^ The risk of relapse is high in patients with TAK. Relapses increase the risk of aggravation of organ ischemia and damage, which in turn affects the prognosis. Thus, long-term follow-up is warranted. EULAR recommendations for the management of LVV stated that for patients with active disease should be followed up every 1–3 months, and then every 3–6 months afterwards.^[110]^ In each visit, disease activity and organ damage assessment which is the basis for adjusting subsequent treatment plans, should be carried out. Image examinations are mainly adopted for disease activity and/or structural damage evaluation. The modalities and frequency of image examinations should be based on the patient’s condition.^[62,76]^ Some adverse reactions are caused by long-term use of GC and DMARDs. Therefore, during each follow-up visit, patient should be monitored for the adverse reactions and complications caused by GCs or csDMARDs, including infection, diabetes, hypertension and osteoporosis. Treatment should be initiated once the complications are identified.

Self-management of the disease by patients themselves is beneficial for controlling disease activity and improving long-term prognosis. Patients should be educated about the nature of the disease and the typical symptoms or signs related to active disease of TAK. Healthy lifestyle can not only prevent or delay the onset of complications but also reduce the risk of cardiovascular events. Healthy diet, restrict daily sodium intake below 5 g, consumption of fresh vegetables and fruits, abstaining tobacco and alcohol intake, controlling body weight, doing exercise, having enough sleep and keeping a positive mood are important to reduce cardiovascular complications. For those with poorly controlled hypertension or severe hypertension, relax is encouraged. They can engage in low-intensity exercise, such as Tai Chi and walking, *etc*.

### Question 11: How to Manage Pregnancy of Patients with TAK?

**Recommendation 11: Patients with TAK are allowed to prepare for pregnancy if their disease has been in remission for at least 6 months, no vital organs damage and the potentially teratogenic drugs have been withdrawn for the required period. Pre-pregnancy consultation and risk assessment should be completed before pregnancy (2C). For pregnant patients, multidisciplinary follow-up and close monitoring is mandatory. Fetal growth and development should be closely monitored. Complications should be identified and managed in a timely manner. The treatment should be adjusted according to the patients’ condition (2C)**.

Fertility of patients with TAK is not affected by the disease in general, but multiple cohort or case-control studies suggested that the disease itself could increase the incidence of pregnancy complications and would negatively impacted pregnancy outcomes.^[111,112,113,114,115,116,117,118,119^] A study that included 379 pregnancies in 294 patients showed that 35% pregnancies experienced hypertension, 30% pregnancies were terminated by caesarean section, 16% pregnancies ended with premature birth, 15% pregnancies had fetal growth restriction (FGR), 12% pregnancies had fetal loss, and 7% of patients had therapeutic abortions. These adverse pregnancy outcomes are much higher than the general population.^[114]^ A study that summarized 104 pregnancies in 84 TAK patients in China showed that 4% of the patients developed gestational hypertension, 20% of the patients had pre-eclampsia, 64% of the patients terminate the pregnancies by cesarean section, 17% of patients had premature birth, 12% of patients had FGR, and 17% of patients had miscarriage or induced abortion.^[115]^ A case-control study from China comparing 110 pregnancies in 80 TAK patients with 550 pregnancies in healthy women demonstrated that adverse pregnancy outcomes were more frequent in TAK patients than those of healthy women. The most common maternal complication is new-onset or worsen hypertension (18.2% [20/110]), and the most prevalent fetal complication is spontaneous abortion (32.7% [36/110]). Adverse pregnancy outcomes are significantly associated with hypertension (adjusted OR 2.67 [95% CI, 1.02–6.98]), renal artery involvement before pregnancy (adjusted OR 2.87 [95% CI, 1.10–7.51]) and active disease during pregnancy (adjusted OR 11.64 [95% CI, 1.45–93.28]).^[116]^ A retrospective cohort study including 240 pregnancies in 96 TAK patients showed that smoking (OR = 6.15 [1.31–28.8]) and active disease during pregnancy (i.e. NIH score > 1) (OR = 28.7 [7.89–104.7]) were independently associated with obstetrical and maternal complications.^[112]^ Therefore, active disease is an important factor leading to adverse pregnancy outcomes. However, there is currently few evidence on how long is needed to be in disease remission before the patient can be safely preparing for pregnancy. Therefore, the expert panel recommend that the disease should be in remission for at least 6 months before preparing for pregnancy. In general, patients with TAK should be assessed for disease activity, blood pressure, arteries involved, comorbidities and medication used prior to pregnancy. Disease in remission for at least 6 months, without medications incompatible to pregnancy and without vital organs dysfunction are the pre-required conditions for patients with TAK to get pregnant.

Controlling active disease and hypertension before and during pregnancy is critical to improve maternal and fetal outcomes. Maternal complications of TAK during pregnancy include new-onset hypertension, worsen of pre-existing hypertension, arterial stenosis/obstruction, heart failure, cerebral ischemia, transient ischemic attack, end-stage renal disease, aortic aneurysm and aortic dissection.^[112]^ For patients with TAK during pregnancy, multidisciplinary management (at least including rheumatologists and gynecologists) is required to monitor disease activity and fetal growth and development. Antihypertensive agents that can be used during pregnancy include labetalol, nifedipine, methyldopa *etc*. Angiotensin-converting enzyme inhibitors and angiotensin-converting enzyme receptor inhibitors should be avoided during pregnancy.^[120]^ For patients with disease relapse or progression during pregnancy, treatment should be adjusted according to the severity of the disease. Azathioprine, calcineurin inhibitors, or TNF-α inhibitors could be added in combination with GC to control the disease if necessary.^[120]^ Taking low-dose aspirin during pregnancy can reduce the risk of preeclampsia and adverse pregnancy outcomes.^[121,122]^ The medications can be used during pregnancy are shown in Table 5.

**Table 5:** Medication use in TAK patients during pre-pregnancy, pregnancy and lactation

drug	Pre-pregnancy	Pregnancy	Lactation
Glucocorticoids	+	recommeded and should be maintained at minimum effective dose	+ Should be maintained at the minimum effective dose; if the dose is greater than 20 mg/d, breastfeeding should be avoided within 4 hours after taking the medication and the milk should be discarded
Hydroxychloroquine	+	+	+
Azathioprine	+	+, maximum dose 2 mg/kg/d	+
Cyclosporin A	+	recommeded and should be maintained at minimum effective dose	+
Tacrolimus	+	+ and should be maintained at minimum effective dose	+
Methotrexate	Discontinue for at least 3 months before pregnancy	—	—
Cyclophosphamide	Clear risk of teratogenicity Discontinue for 3 months before pregnancy	—	—
Mycophenolate mofetil	Clear risk of teratogenicity Discontinue for 3 months before pregnancy	—	—
Leflunomide	Clear risk of teratogenicity. Discontinue for 2 years Or Discontinue for 6 months after taking cholestatic amine for 11 consecutive days (8 g, tid)	—	—
Tocilizumab	Discontinue for 3 months before pregnancy	—	—
Infliximab	+	+	+
Adalimumab	+	+	+
Golimumab	—	—	—
Certolizumab pegol	+	+	+
Rituximab	Discontinue for 6 months before pregnancy	Current evidence does not suggest an increased risk of teratogenicity. rituximab can be used in early pregnancy under special circumstances. Avoid using it in the middle and late stages of pregnancy to prevent risks such as fetal B cell deficiency	—
Intravenous immunoglobulin	+	+	+
Aspirin	+, dose ≤100 mg	+, dose ≤100 mg	+, dose ≤100 mg
Clopidogrel	+	+	+
Rivaroxaban	Discontinue for 3 months before pregnancy	—	+
Low-molecular-weight heparin	+	+	+
Fondaparinux sodium	+	+	+
Warfarin	Discontinue for 3 months before pregnancy	—	+
ACEi/ARBs	Discontinue when pregnancy is confirmed	—	+
Nifedipine	+	+, < 90 mg / day	+
Amlodipine	+	+	+
Labetalol	+	+	+
Methyldopa	+	+	+

+: recommended; -: not recommended.

**Figure 1:**
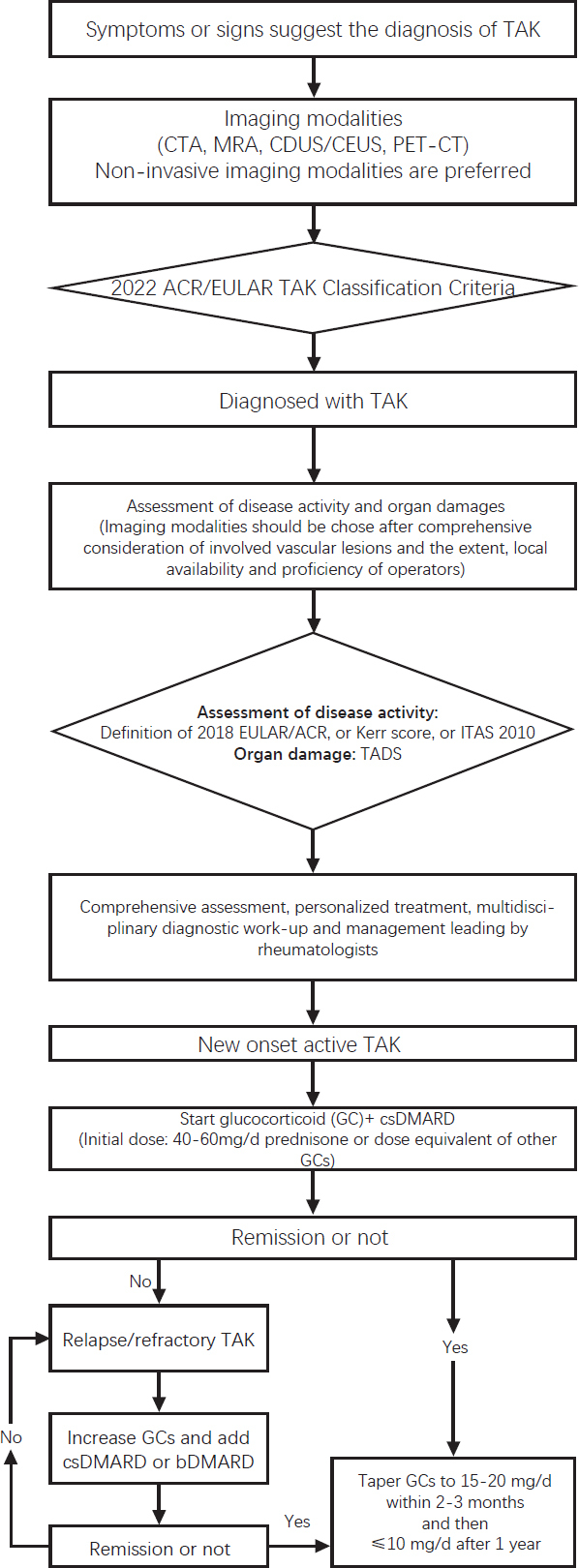
Algorithm for diagnosis and treatment of Takayasu’s arteritis. TAK, Takayasu’s arteritis; CTA, computed tomography angiography; MRA, magnetic resonance angiography; CDUS/ CEUS, color Doppler ultrasound/contrast-enhanced ultrasound; PET-CT, pistron emission tomography-computed tomography; ACR/EULAR, American College of Rheumatology/ European League Against Rheumatism; TADS, Takayasu’s arteritis Damage Scores; ITAS2010, India Takayasu Clinical Activity Score; DMARD, disease modifying antirheumatic drugs.

The guideline is depicted in a simple algorithm presented in Figure 1.

TAK is the most common LVV in adults in China. It can lead to stenosis, occlusion or aneurysm of the aortic arch and its major branches. TAK can cause damage to vital organs such as kidney, heart, brain and lung. Early diagnosis and standardized treatment are crucial to reduce the risk of vital organ damage, reduce complications, as well as improve long-term prognosis and survival. This guideline not only refers to internationally published guidelines but also is evidence-based and consistent with the clinical practice in China. Therefore, this guideline is feasible and may play an important role in improving the standardization of TAK management in China and thus benefit Chinese patients with TAK.
